# Unexpected carbon utilization activity of sulfate-reducing microorganisms in temperate and permanently cold marine sediments

**DOI:** 10.1093/ismejo/wrad014

**Published:** 2024-01-10

**Authors:** Xiuran Yin, Guowei Zhou, Haihua Wang, Dukki Han, Mara Maeke, Tim Richter-Heitmann, Lea C Wunder, David A Aromokeye, Qing-Zeng Zhu, Rolf Nimzyk, Marcus Elvert, Michael W Friedrich

**Affiliations:** State Key Laboratory of Marine Resource Utilization in South China Sea, Hainan University, 58 Renmin Avenue, Haikou 570228, China; Faculty of Biology/Chemistry, University of Bremen, Leobener Strasse 3, Bremen D-28359, Germany; MARUM, Center for Marine Environmental Sciences, University of Bremen, Leobener Strasse 8, Bremen D-28359, Germany; Max Planck Institute for Marine Microbiology, Celsiusstrasse 1, Bremen D-28359, Germany; State Key Laboratory of Marine Resource Utilization in South China Sea, Hainan University, 58 Renmin Avenue, Haikou 570228, China; School of Resources and Environmental Engineering, Anhui University, 111 Jiulong Road, Hefei, Anhui 230601, China; State Key Laboratory of Marine Resource Utilization in South China Sea, Hainan University, 58 Renmin Avenue, Haikou 570228, China; Faculty of Biology/Chemistry, University of Bremen, Leobener Strasse 3, Bremen D-28359, Germany; College of Urban and Environmental Sciences, Peking University, No. 5 Yiheyuan Road, Beijing 100871, China; Department of Marine Bioscience, Gangneung-Wonju National University, 7 Jukheon-gil, Gangneung-si 25457, Republic of Korea; Faculty of Biology/Chemistry, University of Bremen, Leobener Strasse 3, Bremen D-28359, Germany; Max Planck Institute for Marine Microbiology, Celsiusstrasse 1, Bremen D-28359, Germany; Faculty of Biology/Chemistry, University of Bremen, Leobener Strasse 3, Bremen D-28359, Germany; Faculty of Biology/Chemistry, University of Bremen, Leobener Strasse 3, Bremen D-28359, Germany; Max Planck Institute for Marine Microbiology, Celsiusstrasse 1, Bremen D-28359, Germany; Faculty of Biology/Chemistry, University of Bremen, Leobener Strasse 3, Bremen D-28359, Germany; MARUM, Center for Marine Environmental Sciences, University of Bremen, Leobener Strasse 8, Bremen D-28359, Germany; Faculty of Biology/Chemistry, University of Bremen, Leobener Strasse 3, Bremen D-28359, Germany; MARUM, Center for Marine Environmental Sciences, University of Bremen, Leobener Strasse 8, Bremen D-28359, Germany; Faculty of Geosciences, University of Bremen, Klagenfurter Strasse 2-4, Bremen D-28359, Germany; Faculty of Biology/Chemistry, University of Bremen, Leobener Strasse 3, Bremen D-28359, Germany; MARUM, Center for Marine Environmental Sciences, University of Bremen, Leobener Strasse 8, Bremen D-28359, Germany

**Keywords:** sulfate-reducing microorganisms, carbon utilization, fermentation products, RNA-SIP

## Abstract

Significant amounts of organic carbon in marine sediments are degraded, coupled with sulfate reduction. However, the actual carbon and energy sources used *in situ* have not been assigned to each group of diverse sulfate-reducing microorganisms (SRM) owing to the microbial and environmental complexity in sediments. Here, we probed microbial activity in temperate and permanently cold marine sediments by using potential SRM substrates, organic fermentation products at very low concentrations (15–30 μM), with RNA-based stable isotope probing. Unexpectedly, SRM were involved only to a minor degree in organic fermentation product mineralization, whereas metal-reducing microbes were dominant. Contrastingly, distinct SRM strongly assimilated ^13^C-DIC (dissolved inorganic carbon) with H_2_ as the electron donor. Our study suggests that canonical SRM prefer autotrophic lifestyle, with hydrogen as the electron donor, while metal-reducing microorganisms are involved in heterotrophic organic matter turnover, and thus regulate carbon fluxes in an unexpected way in marine sediments.

## Introduction

Marine sediments are the largest organic matter sink on Earth [1]. Mineralization of buried organic matter is driven by the anaerobic microbial food chain, a network of fermenting and anaerobically respiring microorganisms perched below the seafloor [2, 3], which orchestrates the fate of organic compounds as well as the biogeochemical cycling of elements such as carbon, sulfur, nitrogen, iron and manganese [4, 5].

In the anoxic, sulfate-laden layers below the surface of the sediment, i.e. the sulfate reduction zone (SRZ), sulfate-reducing microorganisms (SRM) are one of the most important players that mediate a large fraction of organic matter degradation [6, 7]. Accordingly, SRM are genetically equipped to utilize divergent organic compounds, such as short-chain fatty acids, alcohols, carbohydrates, organohalogens, and aromatics [2, 6, 8]. Among those compounds, organic fermentation products are believed to be the most crucial substrates, the degradation of which is coupled to sulfate reduction as the terminal electron-accepting process in sediments [6, 9]. Fermentation products originating from organic matter degradation such as acetate, lactate, propionate, butyrate, and ethanol are typically present in micromolar concentrations [9-14]. However, organic fermentation products are not under thermodynamic control for potential degraders such as SRM and metal-reducing microorganisms [14-16], and thus it is not clear which microbes are active for low concentration of fermentation products. It is assumed that SRM is one of the most important microbial groups responsible for the degradation of organic fermentation products. For example, multiple SRM, such as representatives of the *Desulfobacteraceae*, *Desulfocapsaceae*, *Desulfosarcinaceae*, and *Desulfovibrionaceae*, can degrade short-chain fatty acids and alcohols [17-20]. Iron-reducing microorganisms *Desulfuromondales* also can use these organic substrates [21]. Sulfate reduction and iron reduction can co-occur in the SRZ of marine sediments [9, 22-25]; however, how fermentation products can be degraded by these microorganisms is not well studied *in situ*.

SRM have been enriched [15, 26-31] and isolated in pure culture [17, 18] from many different sediments, but using concentrations of fermentation products in the mM range, which is much higher than typically encountered *in situ* [9-14]. Apparently, enrichments and pure cultures amended with a high level of fermentation products challenge whether these microorganisms are relevant *in situ*. Detecting the activity modes of SRM in sediments rather than in enrichments or cultures is key to understand their physiological features in the environment as well as their role in elemental cycling. To date, however, much of the work on active SRM has focused on enrichments with known limitations regarding its environmental relevance. Only few studies attempting to capture *in situ* conditions suggest that the well-known SRM of the family *Desulfobacteraceae* can use both, acetate or H_2_ [32-34]. On the other hand, significant discrepancies between sulfate reduction rate and acetate oxidation rate have been reported. Such imbalanced ratios of sulfate reduction and acetate oxidation rates between 1:4 and 20:1 [16, 35-37] suggest that either SRM might use other fermentation products, or other microorganisms present in sediments participate in fermentation product degradation [16, 18, 21, 25, 32, 38-40]; however, the active microbes are not well linked to specific processes when rates are measured.

Given the apparent knowledge gap in understanding the role of SRM in fermentation product degradation in anoxic marine sediments, the following questions require investigation: (i) which fermentation products are utilized by SRM in the SRZ of marine sediment and (ii) what is the impact on biogeochemical cycling therein. We hypothesize that SRM and other microorganisms occupy different but complementary niches for fermentation product utilization. Thus, in the SRZ, the oxidation of organic (e.g. acetate, propionate, butyrate, lactate, ethanol) and inorganic fermentation products (e.g. H_2_) is driven by different guilds of microorganisms and have different trophic categories among these microbes, respectively. However, the low *in situ* concentrations of fermentation products in sediments are difficult to mimic in incubations because such low concentrations challenge the sensitivity of detecting and identifying active SRM without enrichment. To test the hypothesis and overcome the technical limitation, we used the highly sensitive RNA-based stable isotope probing (RNA-SIP) [41] approach with low, close to *in situ* concentrations of multiple organic fermentation products (max. 30 μM) in sediment incubations. RNA-SIP is an ultra-sensitive technique with a threshold below 0.001% of fully ^13^C-labeled nucleic acids [41, 42]. In combination with metagenomic analysis, our findings reveal novel features of fermentation product degradation in the SRZ regarding to carbon cycling in marine sediments.

## Materials and methods

### Sampling and incubation setup for stable isotope probing

**Figure 1:**
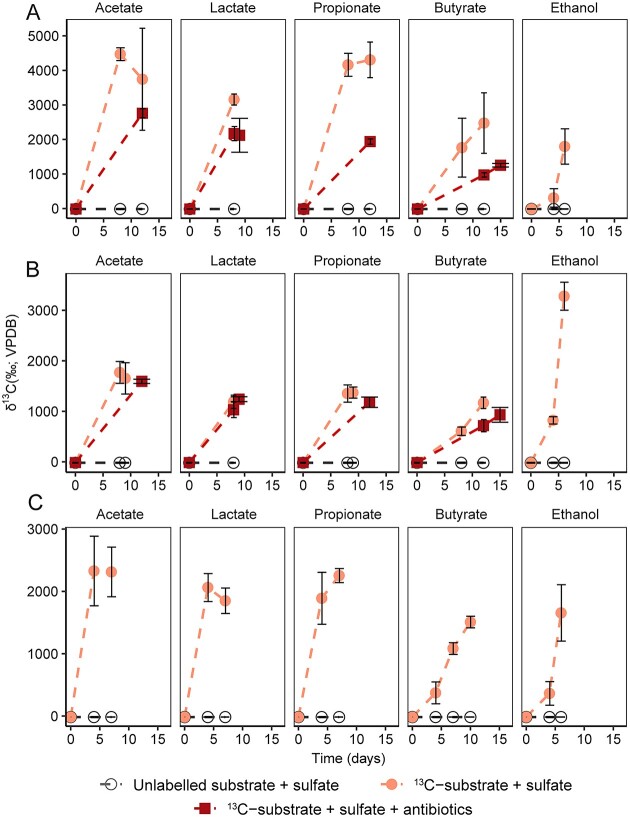
Turnover of fermentation products in SIP incubations; development of δ^13^C-values of headspace CO_2_ in incubations amended with low concentration of fermentation products using Helgoland mud (A), Cumberland Bay (B), and Hornsund fjord sediment (C) (*n* = 3, error bar = SD); VPDB: The Vienna Peedee belemnite; samples for SIP analysis were harvested after the last time point.

Sediments used in this study were sampled from Helgoland mud area (North Sea; 54°05.23′N, 007°58.04′E; RV HEINCKE cruise in 2017; water depth: 27.9 m), Cumberland Bay (South Georgia; 54°15.899′S, 36°26.248′W; M134 cruise in 2017; water depth: 253 m), and Hornsund fjord (Arctic Svalbard; 76°59.325′N, 16°18.320′E; R/V Helmer Hanssen cruise in 2019; water depth: 115 m). Sediment gravity cores were kept at 4°C on board, then the cores were cut in 25 cm sections and stored at 4°C in 2.6-l jars with anoxic artificial sea water and headspace of N_2_. The information of sediment sampling and geochemical profiles were described in the previous studies [43-45]. Slurry incubations were set up with sulfate-rich sediments from the top layers of Helgoland mud area (16–41 cm), Cumberland Bay (14–39 cm), and Hornsund fjord (0–15 cm). Sediments were homogenized with artificial water (w: v = 1: 4, 50 ml; 26.4 g l^−1^ NaCl, 11.2 g l^−1^ MgCl_2_·6H_2_O, 1.5 g l^−1^ CaCl_2_·2H_2_O, and 0.7 g l^−1^ KCl) and filled in sterile 120-ml serum bottles, which were sealed with butyl rubber stoppers. The slurry was vacuumed three times for 3 min in order to remove O_2_ introduced during incubation setup, and headspace of culture was flushed with N_2_ at 1.5 atm as described previously [46]. For improved isotope labelling, slurries were preincubated for 10 days at 10°C to deplete organic substrates, O_2_ and nitrate remaining in the original slurry [15]. Thus, O_2_ was not introduced into incubations in order to mimic the anoxic condition of the sediment used for incubations [44, 47, 48]. After preincubation, all the slurries were amended with low concentrations (60 μM carbon) of fully ^13^C-labeled (99%) organic fermentation products (acetate: 30 μM; propionate: 20 μM; lactate: 20 μM; butyrate: 15 μM and ethanol: 30 μM; provided by Cambridge Isotope Laboratories, Tewksbury, MA). For the inorganic fermentation products, i.e. H_2_/CO_2_, ~100 μM H_2_ in slurry was transferred (15% of H_2_ in the headspace gas given its very low solubility [49]), and 10 mM ^13^C dissolved inorganic carbon was added. All incubations were amended with 18 mM sulfate. Since without amendment of substrate during preincubation will not trigger strong bacterial community shift [50], microbial activity will be identified when ^13^C-labeled substrates were utilized. In order to prove that SRM were present and had ability to degrade organic fermentation products, five antibiotics with the concentration of 50 mg l^−1^ (streptomycin, ampicillin, kanamycin, vancomycin and D-Cycloserine) for each were amended to potentially inhibit the activity of metal-reducing bacteria in incubations using one temperate (Helgoland mud) and one permanently cold (Cumberland Bay) sediments. SRMs such as *Desulfovibrionaceae* and *Desulfobacteraceae* are able to resist antibiotics [51-56]. A parallel set of controls containing unlabeled substrates was also conducted. All slurries were incubated at 10°C in order to have a better comparison among different sediments. After 6–15 days, incubations were stopped based on the development of δ^13^C values of CO_2_ in headspace, which was measured as described previously [57] (see Fig. 1 for the details of the incubation time). For incubations with inorganic fermentation product, i.e. H_2_/CO_2_, samples were harvested after 17 days. To identify the ability of glucose degradation by SRM, 10 μM ^13^C-glucose (i.e. 60 μM carbon) was amended into the SIP incubations, a same setup with the study for organic fermentation products degradation. In order to reveal metal reduction and avoid effect of sulfate, deep sediment from methanic zone (Helgoland mud area: 95–120 cm [58]) was used for SIP incubation setup using organic fermentation products.

### Isopycnic centrifugation, gradient fractionation

For RNA-SIP analysis, RNA was extracted from slurries as described previously [59, 60]. Isopycnic centrifugation and gradient fractionation were employed to separate ^13^C-labeled from unlabeled RNA. Briefly, in order to obtain enough RNA for SIP, we combined RNA from biological replicates (n = 3). About 500–1000 ng of RNA was loaded with formamide (240 μl), cesium trifluoroacetate solution (6 ml, CsTFA, GE Healthcare, Buckinghamshire, UK), and gradient buffer solution. RNA was density separated by an Optima L-90 XP ultracentrifuge (Beckman Coulter, Brea, CA). At the same time, a mixture of fully ^13^C-labeled and unlabeled RNA from *Escherichia coli* was used as standard during density separation for defining heavy and light gradient fraction density ranges. After centrifugation at 124 000*g* at 20°C for 65 h, a total of 14 fractions (~ 410 μl) were collected from each sample. Complementary DNA (cDNA) was then obtained from reverse transcription of RNA using GoScript reverse transcription kit (Promega, Madison, WI). Combination of cDNA from fraction 4 and 5 (heavy), 6 and 7 (middle), 8 and 9 (light) and 10 and 11 (ultra-light) was performed for 16S rRNA sequencing, respectively. RNA quantification was conducted using Quanti-iT RiboGreen (Applied Biosystems, Foster City, CA). SIP fractions including ^13^C-labeled RNA were defined by standardization with RNA of fully labeled and unlabeled RNA standards from *E. coli*.

### 16S rRNA gene sequencing

Polymerase chain reaction (PCR) was performed with barcoded bacterial primer pair (Bac515F: 5′-GTGYCAGCMGCCGCGGTAA-3′; Bac805R: 5′-GACTACHVGGGTATCTAATCC-3′) [61] using KAPA HiFi HotStart PCR kit (KAPA Biosystems, Cape Town, South Africa). Thermocycling was set as follows: 95°C for 3 min; 35 cycles at 98°C for 20 s, 61°C for 15 s, and 72°C for 15 s; 72°C for 1 min. PCR products were then purified and quantified for library preparation [62]. Amplicons were sequenced through NovaSeq 6000 platform (Illumina, San Diego, USA; 2× 250 bp) at Novogene (Cambridge, UK). The raw reads were analysed according to Hassenrück 2022 [63]. Briefly, barcodes were extracted followed by de-multiplexing and primer clipping using cutadapt (version 2.1). The de-multiplexed reads were then analysed using dada2 (version 1.16.0). In detail, the quality of sequencing reads was checked and then the reads were trimmed, followed by the correction of error estimates and error learning in order to retrieve the final clean reads. The clean reads were then dereplicated and denoised, which were further merged for both forward and reverse reads to obtain the long sequences. The chimera reads were then filtered and the unusual reads below 248 bp or above 256 bp were removed. Taxonomy was assigned using the final reads based on the database SILVA 138 database [64] For each sample, 8000 to 60 000 reads were retrieved for abundance analysis.

### Quantitative polymerase chain reaction

cDNA from the heavy, middle, light and ultra-light fractions was used for qPCR in order to quantify *dsrA* transcripts from RNA-SIP fractions as described previously [25]. Each 20 μl reaction mixture consisted of 10 μl of MESA Blue qPCR Master Mix (Eurogentec, Seraing, Belgium), 400 nM primers, 0.2 mg/ml bovine serum albumin (Roche, Mannheim, Germany), 1 ng DNA templates or 2 μl of cDNA samples. The primers DSR1-F+ (5′-ACSCACTGGAAGCACGGCGG-3′ [65]) and DSR-R (5′-GTGGMRCCGTGCAKRTTGG-3′ [66]) were used for qPCR, which are well-designed and have been widely used to identify marine SRM [25, 67, 68]. The qPCR protocol comprised an initial denaturation for 5 min at 95°C and 40 cycles amplification (95°C for 30 s, 60°C for 30 s and 72°C for 40 s). The detection thresholds were above 100 gene copies with an efficiency of 90%–110%.

### Metagenomic analysis

Metagenomic sequencing on the Hiseq 4000 platform (2 × 150 bp) at Novogene (Cambridge, UK) was performed using DNA extracts from the original samples collected from Helgoland mud area (16–41 cm, 50–75 cm, and 222–238 cm) and Cumberland Bay sediments (15 cm, 225 cm, and 975 cm) with different depths, as well as a variety of enrichments using the sediments from the three sites (see Supplementary Table S1). Fourteen samples were used for metagenomic sequencing, with 440 million final clean reads. For metagenomic analysis, the raw reads were analyzed based on the Metawrap package (1.2.1) [69]. Briefly, quality checked reads were trimmed and then assembled using Megahit (1.1.3) with the default settings [70]. Scaffolds (>1000 bps) were binned using a combination of MaxBin2 (2.2.6), CONCOCT (1.0.0), and metaBAT2 (2.12.1). The quality of the bins was improved by remapping the raw reads using short-read mapper BWA (0.7.17) and re-assembled using SPAdes (3.13.0). The completeness and contamination of MAGs were estimated by CheckM2 (0.1.3). Taxonomic classifications of archaeal MAGs were based on GTDB database (0.3.3) (Supplementary Table S1) [71]. The MAGs with middle (≥50% and <10% contamination) and high (>90% complete with <5% contamination) quality according to MIMAG standards [72] were selected for annotation (Supplementary Table S1). Protein-coding regions were predicted using Prodigal (version 2.6.3) [73]. The Kyoto Encyclopedia of Genes and Genomes (KEGG) server (BlastKOALA) [74] (E-value cutoff ≤1e-5), eggNOG-mapper (5.0.0) [75] (E-value cutoff ≤1e-5), InterProScan tool (5.44–79.0) [76] (E-value cutoff ≤1e-10), and mmseq2 (10.6d92c) versus NCBI-nr database searched on April 2020 (E-value cutoff ≤1e-5) were used to annotate the protein-coding regions.

### Phylogenetic analyses

The concatenated set of 71 ribosomal protein genes based on a previously published hidden Markov Model profile [77] were used for phylogenetic analyses in Anvi’o (6.1) [78]. Maximum-likelihood trees were built using IQ-TREE (1.6.12) [79] with the best-fit model (LG + F + R7) and 1000 times ultrafast bootstrapping. The tree files were edited using the online tool iTOL [80].

Because of the different names among GTDB and Silva databases for Sva1033 (*Desulfuromonadales*), 16S rRNA genes in the MAGs for all analysed *Desulfuromonadales* were extracted by Barrnap (version 0.3, http://www.vicbioinformatics.com/software.barrnap.shtml). The 16S rRNA genes together with references were aligned with SINA Aligner [81]. Maximum-likelihood tree was inferred using RAxML (8.2.11) with rapid bootstrapping and the GTRGAMMA model [82]. In our previous work, we have not found evidence for sulfate-reducing archaeal taxa [83], and thus archaeal analysis was not included in this study.

Amino acid sequences of reductive dehalogenase were used for orthology analysis. Reference sequences with 100 hits were retrieved from NCBI nonredundant protein database by blasting sequences of reductive dehalogenase of SRM obtained from this study. The combined sequences of each protein were filtered and clustered using cd-hit (Version 4.6.8) [84] with cut-off of 70%, which was followed by MAFFT-LINSI (Version 7.455) alignment with default parameters [85] and trimming by BMGE with flags “-t AA -m BLOSUM30” [86]. Un-rooted phylogenetic trees for protein sequence were built with 1000 times ultrafast bootstrapping using IQ-TREE with the best-fit models (LG + R8).

## Results

### Turnover of fermentation products in sediment incubations

RNA-SIP incubations with ^13^C labeled substrates and sulfate were set up in order to investigate the turnover of different fermentation products and the activity of the associated microorganisms. Three different sediments were compared including the temperate site Helgoland mud area (North Sea) and two permanently cold sites from Cumberland Bay (South Georgia, sub-Antarctic) and Hornsund fjord (Svalbard, Arctic). The degradation of ^13^C labeled substrate was monitored by the formation of ^13^CO_2_ in the headspace over time to determine incubation stopping time by avoiding cross-feeding when samples were incubated for too long time (Fig. 1). Delta ^13^C values of CO_2_ increased rapidly to ~1030–4470‰ within 15 days for sediments from all three sites. In Helgoland mud sediment incubations, the addition of antibiotics (to suppress SRM competitors [52-56]) to incubations slowed down the formation of ^13^CO_2_ (~1250–2760‰) compared to those without antibiotics (~2470–4470‰) within 15 days (Fig. 1A). In contrast, antibiotics had a smaller inhibitory effect on the turnover of fermentation products in incubations using Cumberland Bay sediment (Fig. 1B). The chosen incubation times were similar for incubations with and without antibiotics (Fig. 1).

### Differential activities of sulfate-reducing microorganisms and other active microorganisms

**Figure 2:**
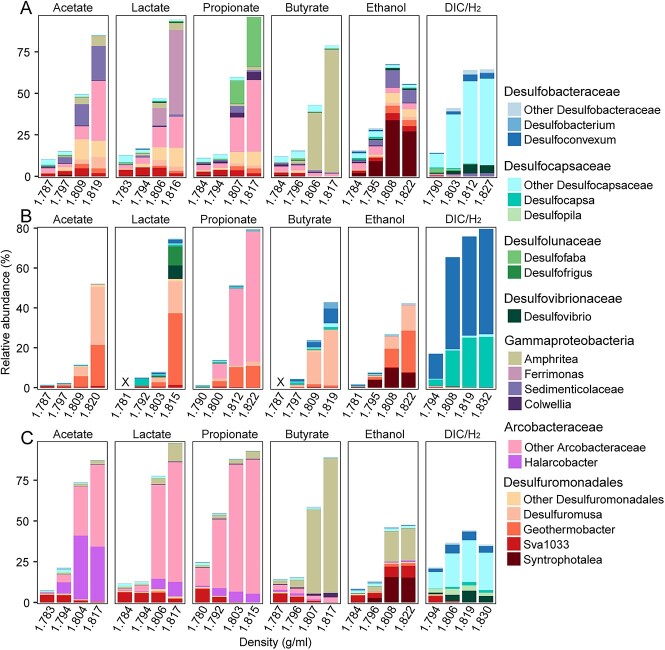
Identification of the active fermentation products degraders using RNA-SIP in incubations amended with ^13^C-labeled substrates and sulfate; relative abundance of 16S rRNA gene sequences of active bacteria fermentation product degraders in total bacteria from RNA-SIP gradient fractions in the Helgoland mud (A), Cumberland Bay (B), and Hornsund fjord sediment (C) incubations; × indicates that cDNA synthesis failed because of insufficient amount of RNA in these fractions; density was indicated as the average density of combined fractions for RNA-SIP samples; for sampling time points, see Figure 1.

Acetate, lactate, propionate, butyrate, and ethanol were used at a concentration of 60 μM carbon, similar *in situ* (15–30 μM dissolved organics carbon), in RNA-SIP incubations to identify those microorganisms actively assimilating labeled substrate during fermentation product degradation. In contrast to the isotopically “light” RNA and unlabeled controls, we recovered several bacterial groups, which were highly abundant in the isotopically “heavy” RNA fractions in their respective incubations (Fig. 2, Supplementary Figs S1 and S2). Most notably, *Desulfuromonadales* members (~15%–50%) were highly active in Helgoland mud and Cumberland Bay sediments (Fig. 2A and B). In addition, members of *Arcobacteraceae* (~18%–65%) were also active when amended with acetate, lactate, and propionate (Helgoland mud) or propionate only (Cumberland Bay) (Fig. 2A and B). We found that those *Desulfuromonadales* groups were likely using iron oxides (lepidocrocite) as electron acceptors in methanic, sulfate-depleted sediments from Helgoland (Supplementary Fig. S3). *Arcobacteraceae* (>50%) also dominated in Hornsund fjord sediment incubations with ^13^C-labeled acetate, lactate, and propionate, whereas active *Desulfuromonadales* (~25%) were found in ^13^C-butyrate incubations of Cumberland Bay sediments. Other notable findings include large active populations of *Sedimenticolaceae* (up to 20%), *Ferrimonas* (up to 50%), and *Amphritea* (up to 74%), which are known as metal reducers as well [40, 87-90], respectively, in specific incubations (Fig. 2A and B). A rather uniform picture emerged from ethanol-amended incubations, in which *Desulfuromonadales* dominated fermentation product degradation in incubations from all three sites. However, canonical SRM were not very active in the incubations with organic fermentation products (acetate, lactate, propionate, butyrate, and ethanol), only showing minor activity in a few incubations (with propionate [Helgoland mud] and lactate[Cumberland Bay]). Instead, *Desulfobacteraceae*, *Desulfocapsaceae*, and *Desulfovibrionaceae* were strongly stimulated (~37–80% in the heavy fraction) when H_2_ and ^13^CO_2_ were amended in the incubations for all the three sites (Fig. 2).

### Sulfate-reducing microorganisms were only utilizing organic fermentation products under inhibition of other microbes

**Figure 3:**
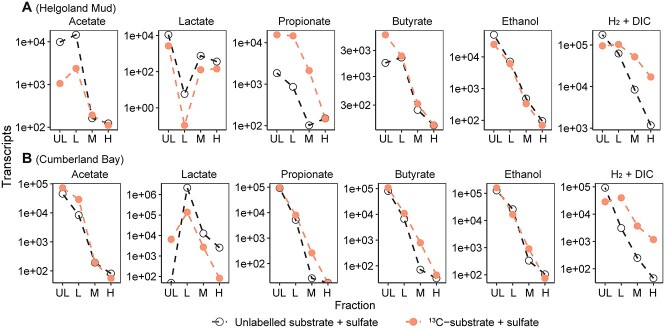
Copies of *dsrA* transcript in different fractions from the RNA-SIP samples; number of transcripts from the heavy (H: 1.815–1.830 g/ml), middle (M: 1.803–1.819 g/ml), light (L: 1.792–1.808 g/ml), and ultra-light (UL: 1.781–1.794 g/ml) fractions of RNA-SIP samples from incubations using Helgoland mud (A) and Cumberland Bay (B) sediment; note that values below 100 copies might be not accurate because the detection threshold was above 100 (see Method).

Since SRM unexpectedly had a minor contribution to organic fermentation product degradation, transcripts of *dsrA* (alpha subunit of the dissimilatory sulfite reductase, a marker gene for sulfate reduction [91]) were quantified within SIP fractions in order to understand the participation of sulfate reduction in incubations with representative cold (Cumberland Bay) and temperate (Helgoland) sediments (Fig. 3). We found that transcripts of *dsrA* from the light fraction were most abundant in any incubation, regardless if ^13^C-labeled or unlabeled substrates were used, while the heaviest fractions always had the lowest amount of *dsrA* transcripts. In contrast to the organic fermentation products, *ds*r*A* abundances in the heavy fractions from inorganic H_2_/DIC were comparatively high, in line with the activity of H_2_/DIC utilization by SRM (Figs 2 and 3).

**Figure 4:**
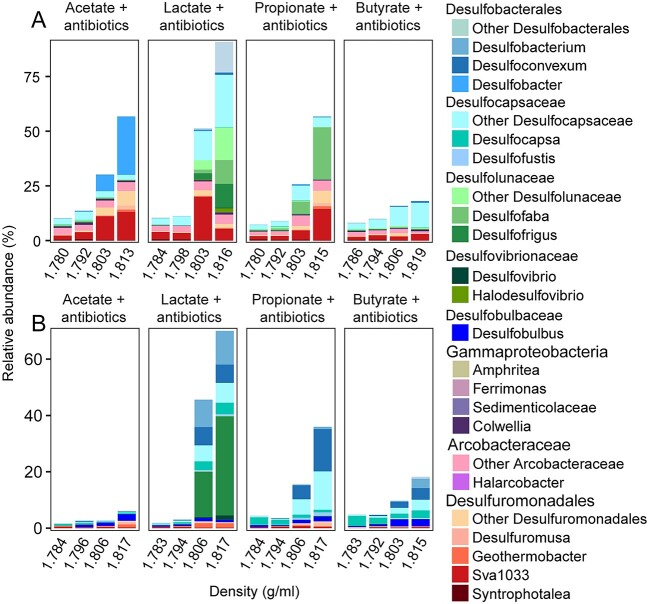
Identification of the active fermentation product degraders using RNA-SIP in incubations amended with ^13^C-labeled substrates, sulfate, and antibiotics; relative abundance of 16S rRNA gene sequences of active bacterial fermentation product degraders in total bacteria from RNA-SIP gradient fractions in the Helgoland mud (A) and Cumberland Bay (B) sediment incubations; Helgoland mud and Cumberland Bay sediments were used as representatives for temperate and permanently cold sediment, respectively; density was indicated as the average density of combined fractions for RNA-SIP samples.

We further checked whether SRM could use these compounds if potential microbial competitors were inhibited. Typical SRM such as *Desulfovibrionaceae* and *Desulfobacteraceae* can resist antibiotics [51-56]. Therefore, multiple antibiotics were added in order to inhibit those organisms which readily used fermentation products in our incubations. In these cases, multiple SRM were actually capable of degrading various fermentation products. In detail, *Desulfobacter* (~27% in heavy RNA fractions) metabolized acetate in Helgoland mud sediment incubations (Fig. 4A). *Desulfobacterales*, *Desulfocapsaceae*, *Desulfolunaceae*, and *Desulfovibrionaceae* (68%–76% of total bacteria) were able to utilize lactate in both sediment types (Fig. 4). *Desulfoconvexum*, *Desulfofaba*, and *Desulfocapsaceae* (29%–34% of total bacteria) could degrade propionate, while *Desulfobacterales*, *Desulfocapsaceae*, and *Desulfobulbus* (14%–18% of total bacteria) participated in the turnover of butyrate, however, on a much lower scale compared to the other treatments (Fig. 4).

### Versatility of carbon metabolic pathways for active fermentation product utilizers and noncanonical sulfate-reducing microorganisms

**Figure 5:**
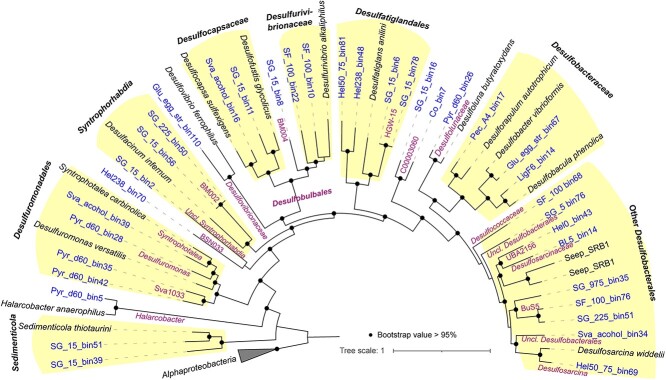
Maximum likelihood tree of 71 concatenated bacterial genes; Uncl.: unclassified; the tree was built using IQ-TREE (1.6.12) with the best-fit model (LG + F + R7) and 1000 times ultrafast bootstrapping; see Supplementary Table S1 for the details of MAG information.

In SIP incubations, we identified the activity of fermentation product degraders and some SRM. In order to have a deeper insight into the genomes of SRM and other fermentation product degraders in sediments, we screened 36 metagenome-assembled genomes (MAGs) with middle to high quality from the original sediments and sediment enrichments (Fig. 5, Supplementary Table S1). These MAGs were affiliated to the active fermentation product degraders including *Sedimenticolaceae*, *Halarcobacter*, Sva1033, *Desulfuromonadaceae Desulfovibrionaceae*, *Desulfocapsaceae*, and *Desulfobacteraceae*, and noncanonical SRM such as BSN033 (class level of *Desulfobacterota*), *Syntrophorhabdia*, *Desulfurivibrionaceae*, *Desulfatiglandales*, C00003060 (order level of *Desulfobacteria*), and other *Desulfobacterales* (Fig. 5, Supplementary Fig. S4, Supplementary Table S1).

**Figure 6:**
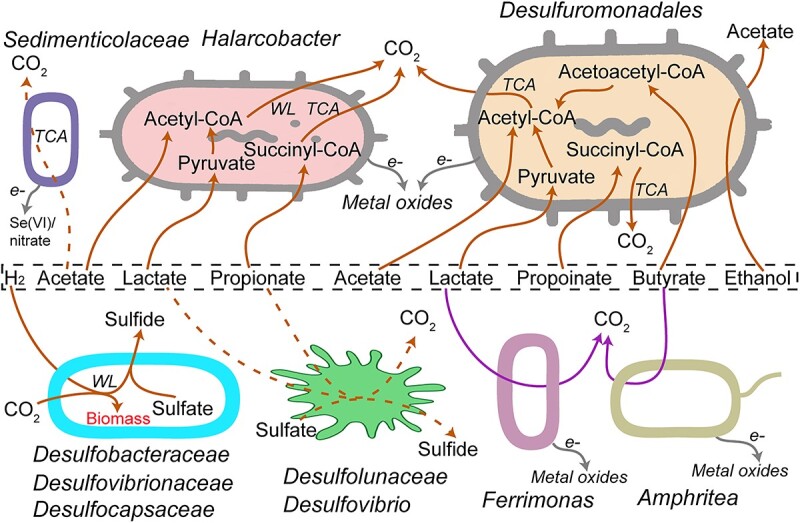
Patterns of fermentation products utilization in SRZ of temperate and permanently cold marine sediments based on SIP and metagenomics; solid and dashed brown lines indicate the strong and weak activity for fermentation product utilization, respectively; see Table S2 for the details of annotated genes; TCA: Citric acid cycle.

Based on metagenomic analysis, pathways involved in the degradation of fermentation products were widely identified in most MAGs of active microbes. In detail, MAGs of *Desulfuromonadales* such as Sva1033 and *Desulfuromonas* included the pathways for acetate, lactate, propionate, and butyrate dissimilation to the corresponding acyl-CoA, which can be oxidized to CO_2_ via the acetyl-CoA pathway or citric acid cycle coupled to iron reduction, but they did not feature genes for H_2_ oxidation (Fig. 6, Supplementary Tables S2, S3). *Halarcobacter* were equipped with pathways for acetate, lactate, and propionate degradation similarly to *Desulfuromonadales* (Fig. 6, Supplementary Table S2). In addition, *Pelobacteraceae* had gene sets for ethanol oxidation to acetate, a known feature of *Pelobacter* spp. (now partly known as *Syntrophotalea* spp.; [92]) such as *Pelobacter acetylenicus* (now *Syntrophotalea acetylenica* [92]) (Fig. 6, Supplementary Table S2). For active SRM, the metagenomic analysis indeed reflected that H_2_/CO_2_ utilization via Wood–Ljungdahl (WL) is a common feature for SRM indicated by the presence of complete WL pathway and hydrogenases including group 1a, 1b, or 1c (hydrogenotrophic respiration using sulfate [93, 94]) in the MAGs of *Desulfobacterales* and *Desulfobulbales* (Supplementary Table S3).

We further checked the metagenomic pathways of noncanonical SRM to predict their potentials for alternative organic carbon degradation pathways. We found that SRM groups including *Desulfatiglandales* and other *Desulfobacterales* harbored the pathways for reductive dehalogenesis and potentials involving in aromatic compound degradation (Supplementary Figs S5 and S6, Supplementary Table S2). Furthermore, multiple MAGs have pathways for glycolysis and fatty acid degradation in SRM including Syntrophorhabdia, *Desulfobulbales*, C00003060, and other *Desulfobacterales* (Supplementary Fig. S4). Such sugar utilization was also indicated from the SIP incubations in which the RNA of *Desulfobacterales* (*Desulfocapsaceae*) was specifically labeled by ^13^C-glucose in the presence of antibiotics (Supplementary Figs S7 and S8).

## Discussion

### Partitioning of fermentation product degradation among canonical fermentation product degraders

Using ultra-high sensitivity RNA-SIP in combination with relevant ^13^C-labeled fermentation products, we found a consistent partitioning pattern in temperate and permanently cold sediments: organic fermentation products were mostly used by known and novel metal-reducing bacteria, whereas SRM were strongly active in using H_2_ autotrophically.

In general, the concentration of organic fermentation product in coastal sediments is low, ranging from nanomolar to few-hundred micromolar [9-14]. In incubations, we used only 60 μM carbon (15–30 μM fermentation products) for SIP and thus, matched closely *in situ* concentrations of organic fermentation products. This in turn is likely avoiding enrichment artifacts originating from irrelevant carbon compound concentrations in incubations and thus, better reflects microbial activities in the studied sediments. We did not detect a strong enrichment of SRM on H_2_/CO_2_ since the abundance of unlabeled *dsrA* was still higher in the light fractions than that of the heavy fractions. Based on this, we found that *Desulfuromonadales* (*Desulfuromusa*, *Geothermobacter*, *Syntrophotalea*, and Sva1033), *Arcobacteraceae*, *Ferrimonas*, *Sedimenticolaceae*, *Amphritea,* and *Syntrophotalea* were the main organic fermentation product utilizers while SRM were incorporating ^13^CO_2_ with H_2_ as electron donor. The former microorganisms are not known as SRM [25, 38, 92], which is corroborated by the absence of genes encoding the dissimilatory sulfate reduction pathway in their MAGs (Supplementary Table S2). As revealed in our studies, *Desulfuromonadales* were iron-reducing bacteria (Supplementary Fig. S3, Fig. 6). Certainly, dissolved Fe(II) and Mn(II) were not detectable due to low concentration of amended substrates in SIP incubations, and abiotic reactions resulting in the formation of insoluble minerals (e.g. siderite) [95]. However, the identified active non-SRM (*Desulfuromonadales* – *Desulfuromusa*, *Geothermobacter*, and Sva1033) here were identified previously in Helgoland mud and Cumberland Bay sediment incubations as iron-reducing microbes (Supplementary Fig. S3) [25, 58]. It is also feasible that *Arcobacteraceae* reduce Fe(III) or Mn(IV) as electron acceptor linked to the oxidation of organic carbon compounds [25, 40, 96, 97]. The other active ^13^C-labeled bacteria, such as *Sedimenticolaceae*, *Ferrimonas*, and *Amphritea*, can utilize metal oxides such as manganese, iron, and selenium oxides or have been identified in incubations amended with metal oxides in several studies [25, 40, 87-90, 98]. Gas exchange in headspace and preincubation of sediments (see Method) ensured that alternative electron acceptors (e.g. traces of oxygen, nitrate) were depleted. In our study, we have used marine sediment from one temperate and two permanently cold sites. The temperate sediment from the Helgoland mud area is characterized by high sedimentation rate resulting in deeply buried iron oxides, which can fuel microbial activity in anoxic sediment layers [58, 99]. In cold sediment from Cumberland Bay (South Georgia, sub-Antarctic) and Hornsund fjord (Svalbard, Arctic), metal oxides are originating from glacier-associated erosion and meltwater, and thus metal oxides such as iron and manganese oxides are present [100, 101]. Thus, the identification of active degraders of fermentation product as known metal reducing microorganisms suggests that sediments contained sufficient amounts of metal oxides as electron acceptors to support their metal-reducing activity, even in deep sediment from the methanic zone (Supplementary Fig. S3) [25, 40].

In our SIP incubations, SRM were not actively degrading and incorporating label from organic fermentation products; this is surprising as sulfate is present at high concentration (15 to 28 mM *in situ*; 18 mM in our incubations) in the upper sediment layer of the studied sediments, and SRM have been identified in this and previous studies at high abundance (Supplementary Fig. S9) [25, 43, 99, 102]. Albeit their limited activity, SRM were still active in incubations but rather using other substrates since *dsrA* transcripts were much more abundant in the light fraction than those from heavy fraction, and thus, were not labeled from ^13^C-organic fermentation products (Figs 2 and 3). Only in the presence of antibiotics, SRM were found to incorporate ^13^C-label from added organic substrates (Fig. 4), ruling out the possibility that amended low concentrations of organic fermentation products were limiting the activity of SRM, and corroborating that the turnover of organic fermentation products is apparently not under thermodynamic control in marine sediments [14-16]. The low *in situ* temperature might trigger the observed partitioning of organic fermentation product degradation among SRM and other microbes: low activity of SRM at temperatures below 10°C; iron reduction in temperate marine sediment was apparently favored at low temperatures (4–10°C) [62], while the optimal temperature for sulfate reduction was found to be above 15°C for cold sediments [15], hinting to a potentially better adapted metabolism of iron-reducing microorganisms at lower temperature. With antibiotics, most metal-reducing bacteria were inhibited and therefore SRM were identified, which further indicates that there was no O_2_ contamination that might inhibit the activity of SRM. In this study, we used temperate and permanently cold sediment and found that these two types of sediments have very similar features for the activity of SRM at relatively low temperate condition. It is still very interesting for the future study to test the ability of SRM for organic fermentation product utilization in high temperature sediments.

Unlike organic fermentation products, the H_2_ concentration is typically under thermodynamic control in marine sediments [15, 49, 103, 104], thus, the hydrogen partial pressure is determined by the free energy available of the energetically most favorable electron accepting process. In sulfate reduction-dominated coastal sediment, hydrogen partial pressures were on similar levels regardless whether metal oxides were added or sulfate was present [103], suggesting that out-competition of SRM by metal reducing microorganisms based on terminal electron acceptor thermodynamics [105] was not operative. Albeit high quality of MAGs, we found a lack of respiratory H_2_-uptake [NiFe]-hydrogenase and incomplete WL pathways in MAGs of Sva1033 and *Desulfuromonas* spp. (Supplementary Tables S1–S3), suggesting that these iron-reducing microorganisms cannot oxidize hydrogen and fix CO_2_. In contrast, SRM were strongly stimulated in SIP incubations with H_2_/^13^CO_2_. Many SRM species in our marine sediment incubations are actually autotrophs capable of fixing inorganic carbon and using H_2_ as electron donor (Fig. 6). Besides primary fermentation, secondary, syntrophic oxidations of organic fermentation products are important sources of H_2_ in marine sediments [2], and H_2_ can contribute up to 75% in electron flow [106]. Based on our study, SRM can indirectly participate in organic matter degradation by interspecies hydrogen transfer interactions during fermentation of macromolecules such as protein, carbohydrates, and cell biomass [33, 37]. Thermodynamically, hydrogen is also a sufficiently strong reductant for CO_2_ fixation in the relevant reactions (oxidation of H_2_: H_2_ = 2e^−^ + 2H^+^; E°′ = −414 mV; reduction of CO_2_ to formate, E°′ = −430 mV; CO_2_ to CO, E°′ = −520 mV, acetyl-CoA and CO_2_ to pyruvate (E°′ = −500 mV) [107]). In fact, some genera affiliated with the family *Desulfobacteraceae* remain lithoautotrophic in the presence of H_2_ when acetate is amended [108], indicating H_2_-based preference for lithoautotrophy in some SRM. In addition, other than H_2_, direct interspecies electron transfer might be another mechanism supporting autotrophy of SRM in environments. For example, *Desulfosarcina*/*Desulfococcus* utilize electrons transferred from their methanotrophic ANME partners for autotrophic growth [109], a syntrophic consortium mediating anaerobic methane oxidation [110]. Although ~100 μM H_2_ in slurry was amended, given that SRM were not active for almost all the common organic fermentation products (acetate, lactate, propionate, butyrate, ethanol) and the substantial overpressure in sediments [111], utilization of H_2_/CO_2_ or interspecies electron most likely reflected the activity of SRM *in situ*. Overall, the SIP results suggested that SRM were of minor importance during organic fermentation product degradation (Fig. 6).

### Beyond fermentation products: non-canonical organic carbon utilization by sulfate-reducing microorganisms

Beyond active SRM, marine sediments inhabit diverse uncultivated SRM (Supplementary Fig. S9), and thereby this leads to the question of what their potential role in carbon degradation in these sediments is. Our SIP incubations amended with glucose suggested that SRM were able to degrade glucose in the presence of antibiotics (Supplementary Fig. S7), and thus they had the ability for glucose uptake into cells and also harbor the complete pathway for glucose degradation (Supplementary Fig. S5). Although the SRM were outcompeted by sugar fermenters when antibiotics were not present (Supplementary Fig. S8), it is still notable that some other carbohydrates might be the substrates for SRM in marine sediments, which is in line with the observation of carbohydrate degradation by SRM in a few studies [112-115]. Apart from glucose utilization, our enrichment incubations and metagenomic analysis also indicated a wider spectrum of substrate utilization by noncanonical SRM than expected, such as halogens (see supplemental discussion), and thus SIP experiments should focus on the carbon utilization versatility of SRM in the future study.

Our study has new implications for the role of SRM on biogeochemical cycling in marine sediment: (i) canonical SRM have limited contribution on the degradation of organic fermentation products at low concentrations in marine sediments, (ii) canonical SRM appear to prefer autotrophic lifestyle using H_2_ oxidation instead of heterotrophy, (iii) many SRM have potentials for utilizing noncanonical carbon compounds. Canonical SRM may actually have an autotrophic lifestyle in environments. In the SRZ, CO_2_ assimilation has been identified in some archaeal groups such as *Lokiarchaeota*, *Bathyarchaeota*, and ANMEs [116-118], while their activities are quite low. It has been recognized that autotrophy is an important lifestyle for sulfur oxidizers with a relatively high activity of bacteria [119]. Based on our findings, we propose that SRM are additional CO_2_ assimilators that have to be considered to regulate carbon fluxes in marine sediments.

## Supplementary Material

Supplemental2_wrad014

Stables_wrad014

## Data Availability

The bacterial MAGs data are available in NCBI database under the project PRJNA678468 with the accession numbers of SAMN32874205 to SAMN32874239. Sequencing data of RNA-SIP samples have been deposited in the Short Reads Archive under the project PRJNA505997 with accession numbers from SAMN32873837 to SAMN32874024. The metagenomic reads sequenced from original sediments have been deposited under the project PRJNA1023477 with accession numbers from SAMN37668301 to SAMN37668306.
