# Supplementary material for: Unexpected carbon utilization activity of sulfate-reducing microorganisms in temperate and permanently cold marine sediments
Source: ISME J. 2024 Jan 10;18(1):wrad014. doi: 10.1093/ismejo/wrad014 (PMC10811731; doi:10.1093/ismejo/wrad014)
Supplement: Supplemental2_wrad014 [file supplemental2_wrad014.pdf]

Unexpected carbon utilization activity of sulfate-reducing microorganisms in temperate and permanently cold marine sediments

Xiuran Yin[1,2,3,4*], Guowei Zhou[1,5*], Haihua Wang[1,2,6*], Dukki Han[7], Mara Maeke[2,4], Tim Richter-Heitmann[2], Lea C. Wunder[2,4], David A. Aromokeye[2], Qing-zeng Zhu[3], Rolf Nimzyk[2], Marcus Elvert[3], Michael W. Friedrich[2,3]

[1]State Key Laboratory of Marine Resource Utilization in South China Sea, Hainan University, Haikou, China

[2]Faculty of Biology/Chemistry, University of Bremen, Bremen, Germany.

[3]MARUM, Center for Marine Environmental Sciences, University of Bremen, Bremen, Germany.

[4]Max Planck Institute for Marine Microbiology, Bremen, Germany

[5]School of Resources and Environmental Engineering, Anhui University, Hefei, Anhui, China.

[6]College of Urban and Environmental Sciences, Peking University, Beijing, China.

[7]Department of Marine Bioscience, Gangneung-Wonju National University, Gangneung 25457, Republic of Korea

[*]Correspondence:

Xiuran Yin, Faculty 02 (Chemistry/Biology) & MARUM, University of Bremen, Leobener Straße 3, D-28359, Bremen, Germany; E-mail: yin@uni-bremen.de; Tel: +49-421-218-63060.

Guowei Zhou, School of Resources and Environmental Engineering, Anhui University, 230601, Hefei, People’s Republic of China. E-mail: gwzhou@ahu.edu.cn; Tel: +86-551- 63861441.

Haihua Wang, College of Urban and Environmental Sciences, Peking University, Beijing, 100871, China; E-mail: wanghaihua@pku.edu.cn.

Supplemental discussion

Non-canonical organic carbon utilization by SRM

Based on metagenomic analysis, the inactive SRM such as *Desulfatiglandales* and *Desulfobacterales* had pathways for multiple carbon compound utilization. For example, more than 10 homologs of reductive dehalogenase genes were identified specifically in group HGW-15 of *Desulfatiglandales* (Fig. S6), indicating that these groups might utilize different organohalides as electron acceptors [1]. Such feature of *Desulfatiglandales* correlated with their distribution in deep sediments with a high abundance where sulfate is depleted (Fig. S9). In addition, *Desulfatiglandales* and *Desulfobacterales* had completed β-oxidation, the pathway for long-chain fatty acid degradation. Overall, it seems that SRM feature a variety of carbon sources and thus future experiments should focus on the carbon utilization versatility of SRM.

Supplemental Figures

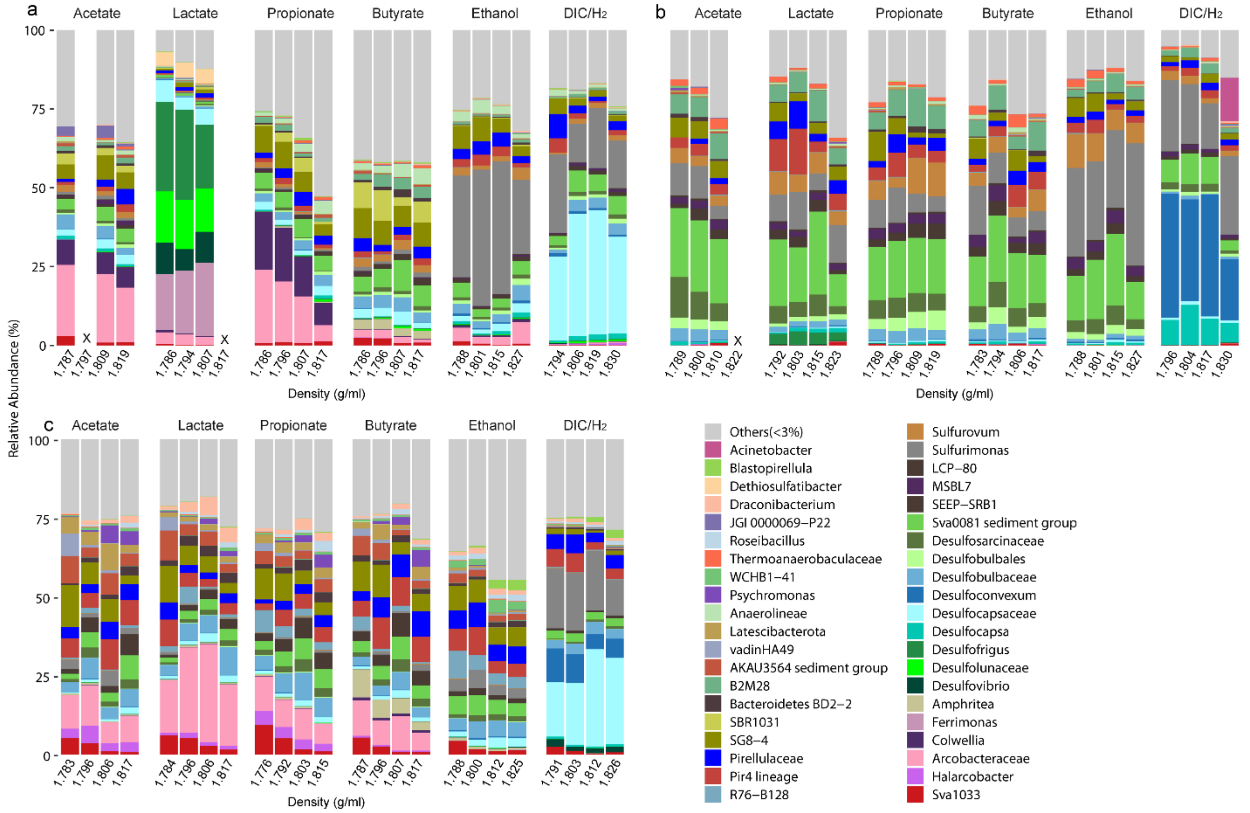

Fig. S1 Distribution of bacterial community among different RNA-SIP fractions from the incubations amended with unlabelled substrates. Relative abundance of 16S rRNA gene sequences of bacteria from RNA-SIP gradient fractions in the Helgoland mud **(a)**, Cumberland Bay (**b**) and Hornsund fjord **(c)** sediment incubations with unlabelled substrates. × indicates that cDNA synthesis failed because of insufficient amount of RNA in these fractions.

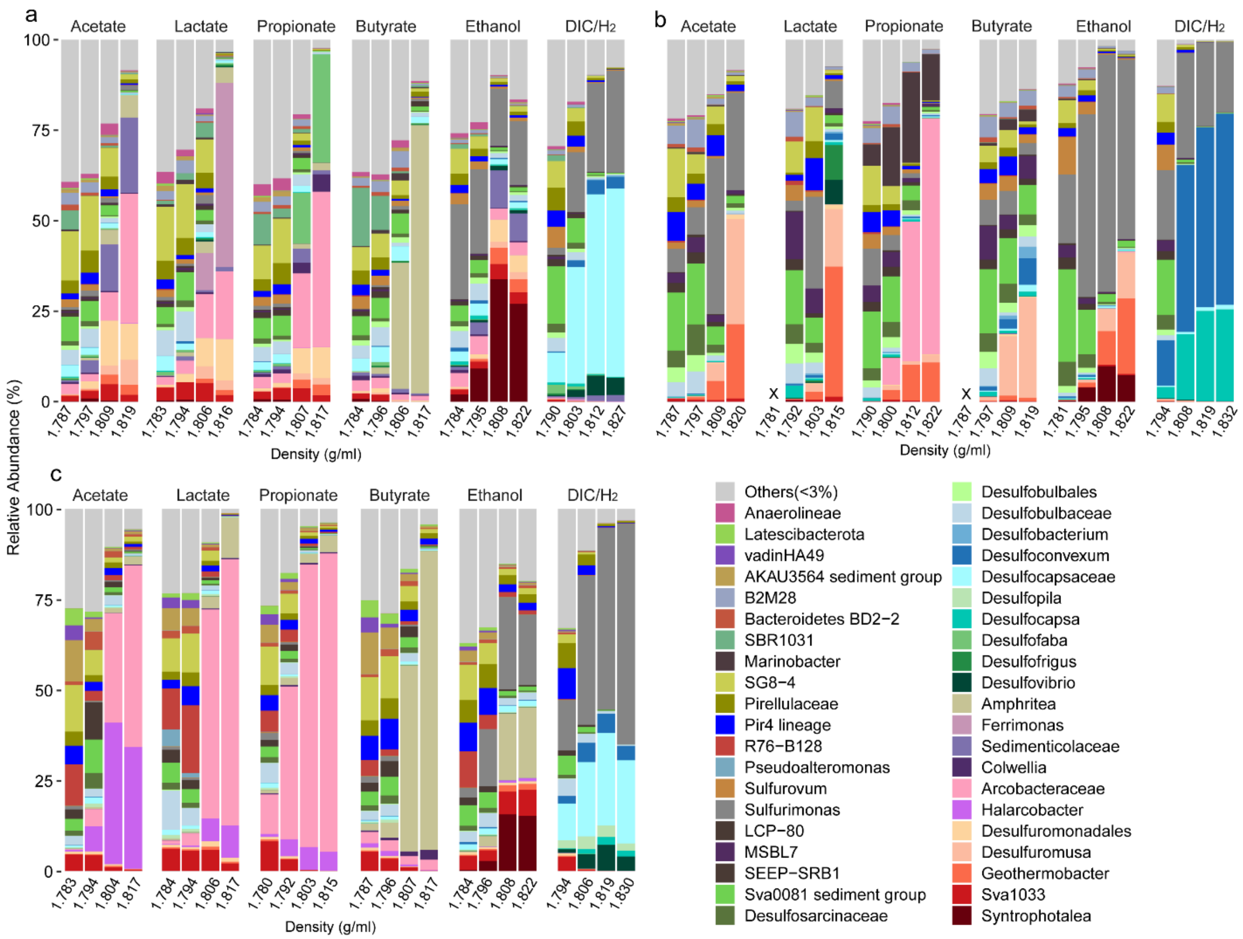

Fig. S2 Distribution of bacterial community among different RNA-SIP fractions from the incubations amended with ^{13}C-labelled substrates. Relative abundance of 16S rRNA gene sequences of bacteria from RNA-SIP gradient fractions in the Helgoland mud **(a)**, Cumberland Bay (**b**) and Hornsund fjord **(c)** sediment incubations with unlabelled substrates. × indicates that cDNA synthesis failed because of insufficient amount of RNA in these fractions.

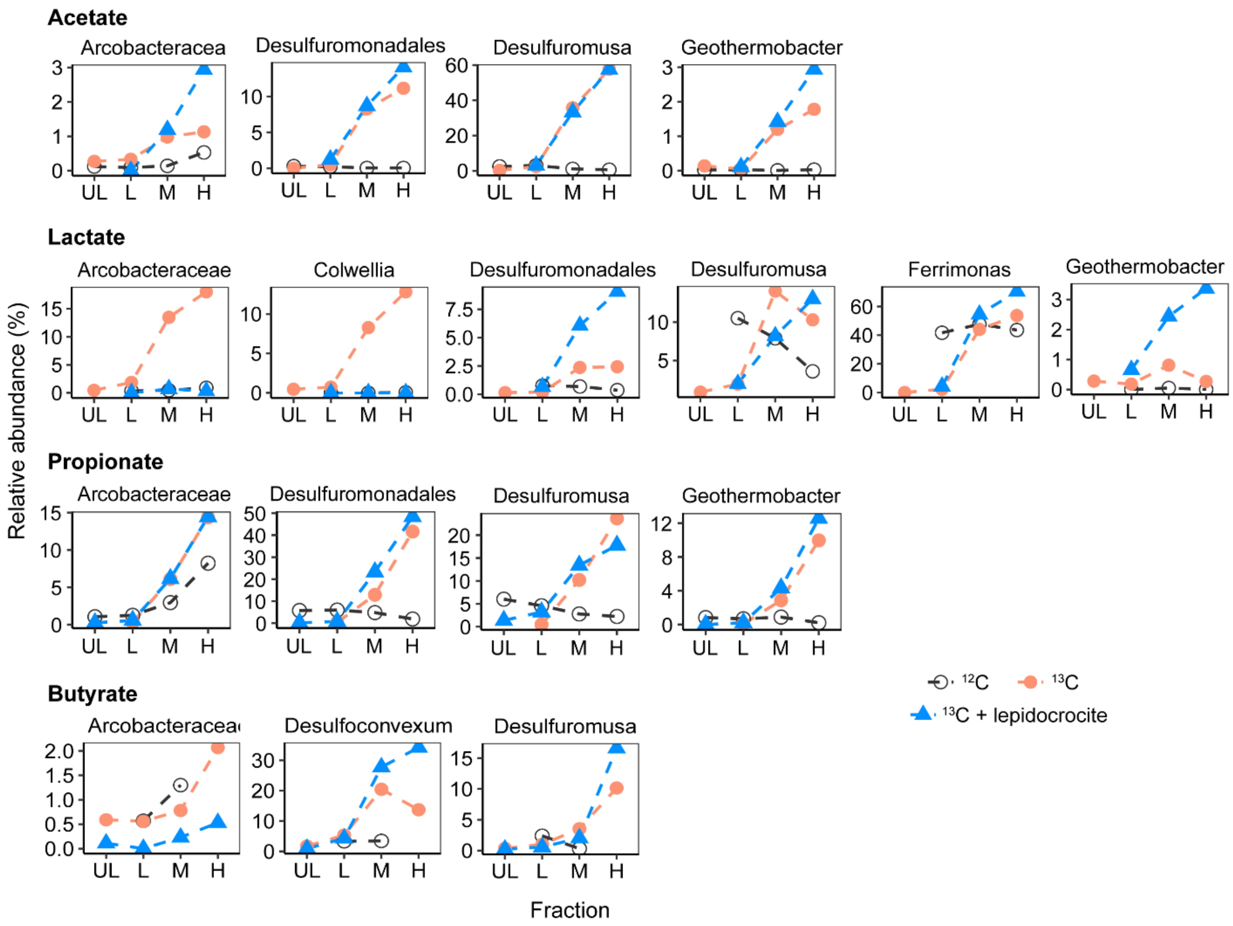

Fig. S3 Relative abundance of active microbes in different RNA-SIP fractions from the incubations using methanic zone sediments (95-120 cm). H: heavy fractions, 1.815-1.817 g/ml; M: middle fraction 1.803-1.806 g/ml; L: light fraction, 1.792-1.796 g/ml and UL: ultra-light fraction 1.783-1.786 g/ml. Sulfate was not amended into the incubations.

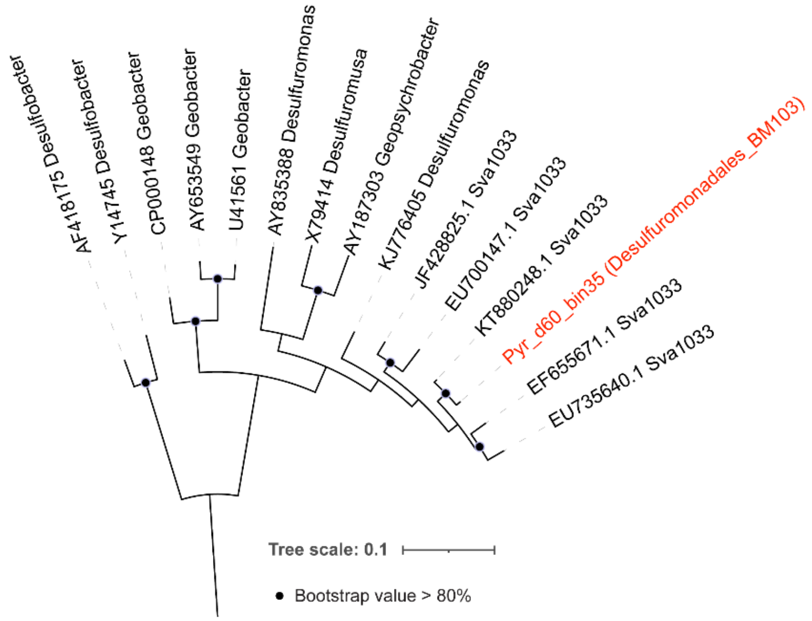

Fig. S4 Classification of MAG BM103 as Sva1033 (see Table S1 for the details of MAG information).

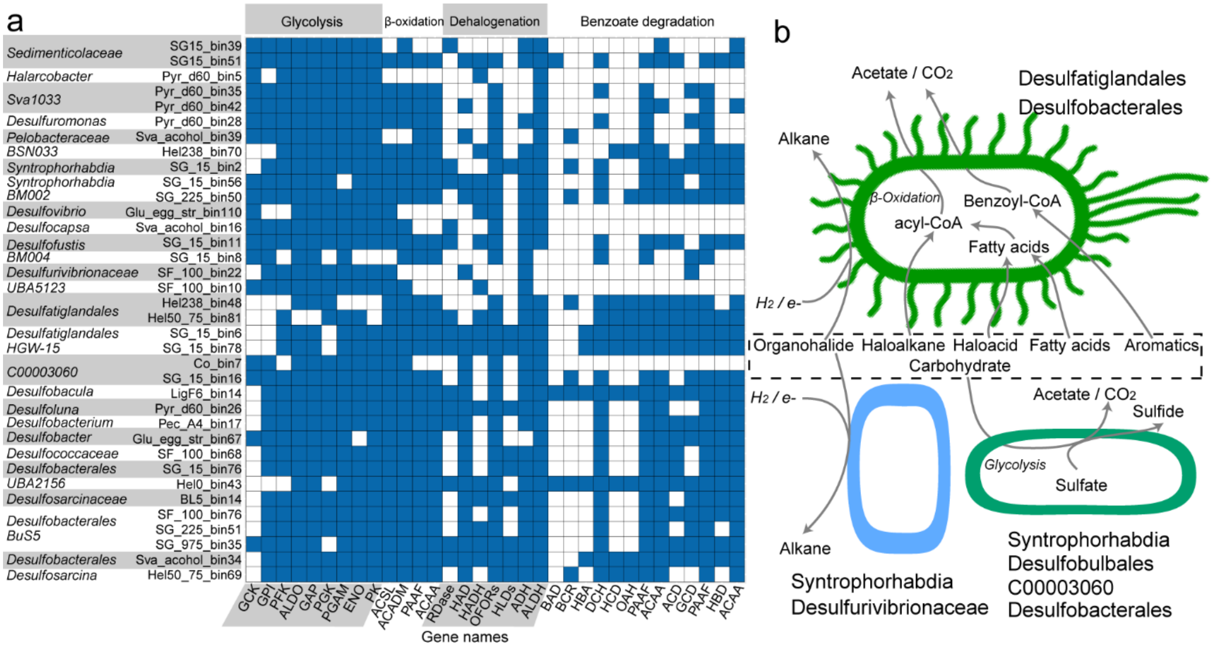

Fig. S5 Metagenomic analysis for the inactive SRM. (a) Annotation of carbon degradation pathways in MAGs obtained from original sediment and incubations from Helgoland mud, Cumberland Bay and Hornsund fjord sediment. (b) The proposed pathways for carbon utilization by inactive SRM in sediments. Blue and white indicate the presence and absence of genes in MAGs, respectively. GCK: glucokinase; GPI: glucose-6-phosphate isomerase; PFK: 6-phosphofructokinase; ALDO: fructose-bisphosphate aldolase; GAP: glyceraldehyde-3-phosphate dehydrogenase; PGK: phosphoglycerate kinase; PGAM: phosphoglyceromutase; ENO: enolase; PK: Pyruvate kinase; ACSL: long-chain-fatty-acid-CoA ligase/acyl-CoA synthetase; ACADM: acyl-CoA dehydrogenase; PAAF: enoyl-CoA hydratase/3-hydroxyacyl-CoA dehydrogenase; ACAA: acetyl-CoA acyltransferase; RDase: reductive dehalogenase; HAD: haloacid dehalogenase; HADH: hydroxyacid dehydrogenase; OFORs: 2-oxoacid ferredoxin oxidoreductase; HLDs: haloalkane dehalogenase; ADH: alcohol dehydrogenase; ALDH: aldehyde dehydrogenase; BAD: benzoate-CoA ligase; BCR: benzoyl-CoA reductase; HBA: 4-hydroxybenzoyl-CoA reductase; DCH: cyclohexa-1,5-dienecarbonyl-CoA hydratase/enoyl-CoA hydratase; HCD: 6-hydroxycyclohex-1-ene-1-carbonyl-CoA dehydrogenase; OAH: 6-oxocyclohex-1-ene-1-carbonyl-CoA hydratase; ACD: glutaryl-CoA dehydrogenase; GCD: glutaconyl-CoA decarboxylase; HBD: 3-hydroxybutyryl-CoA dehydrogenase. See Table S2 for the details of annotated genes.

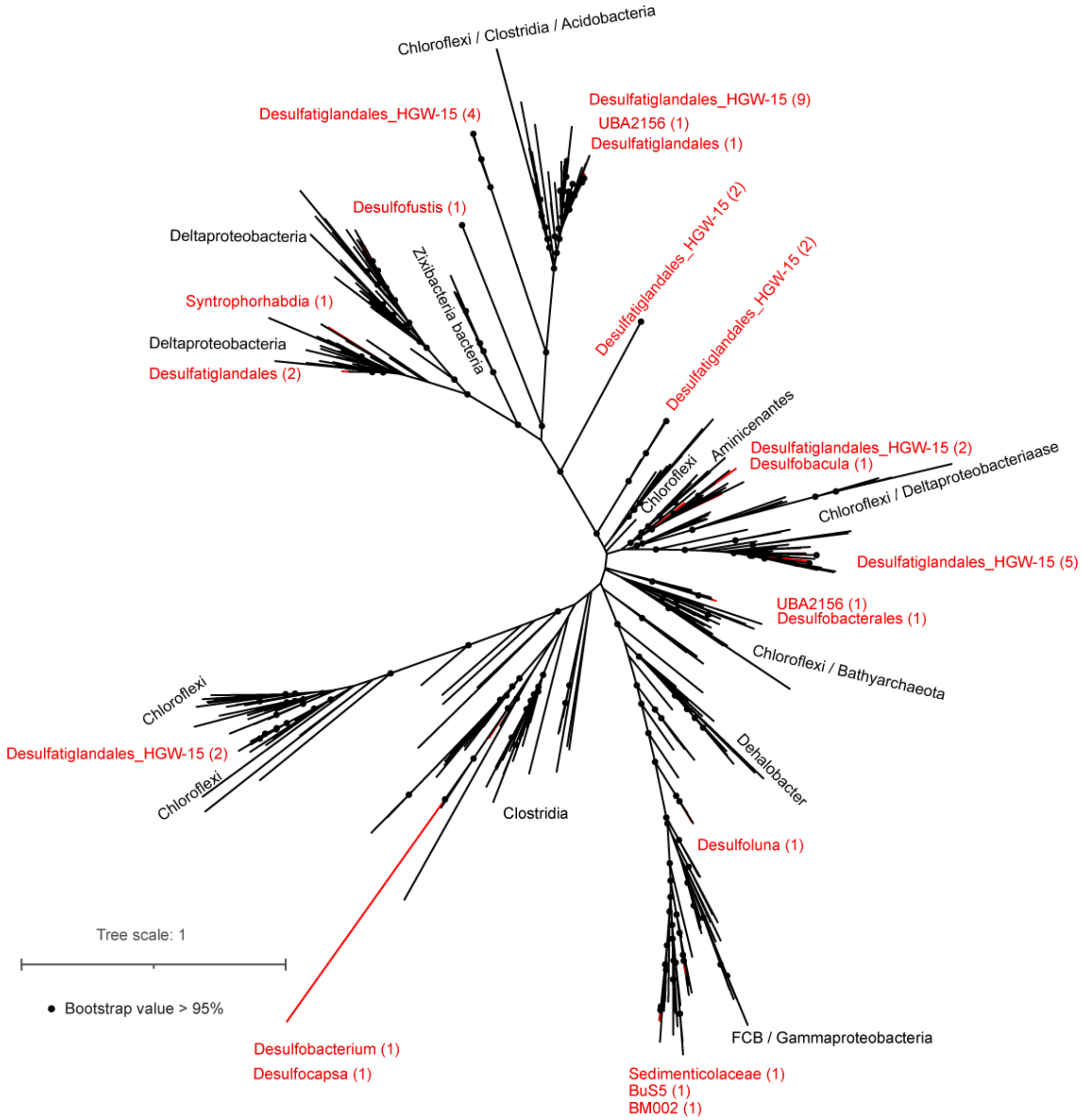

Fig. S6 phylogeny of reduction dehalogenase for sulfate reducing bacteria (see Methods for the details of the tree calculation).

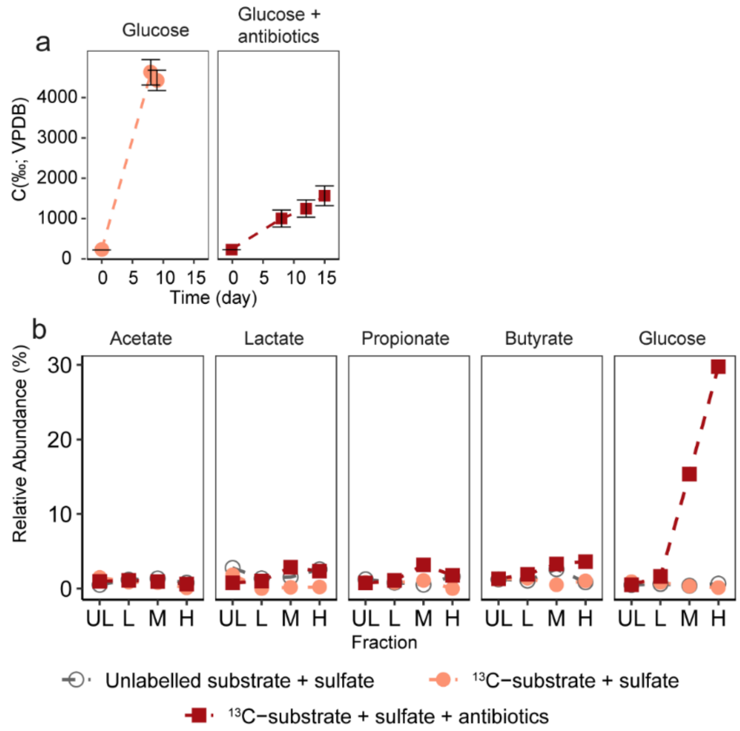

Fig. S7 Identification of *Desulfocapsaceae* for glucose degradation in SIP incubations. **(a)** Development of δ^{13}C-values of headspace CO_2 in incubations amended with low concentration of FPs using Helgoland mud sediment. **(b)** Relative abundance of 16S rRNA gene sequences of *Desulfocapsaceae* in total bacteria from RNA-SIP gradient fractions. Similar with the other treatments, 60 µM carbon, i.e., 10 µM glucose was amended for SIP incubations.

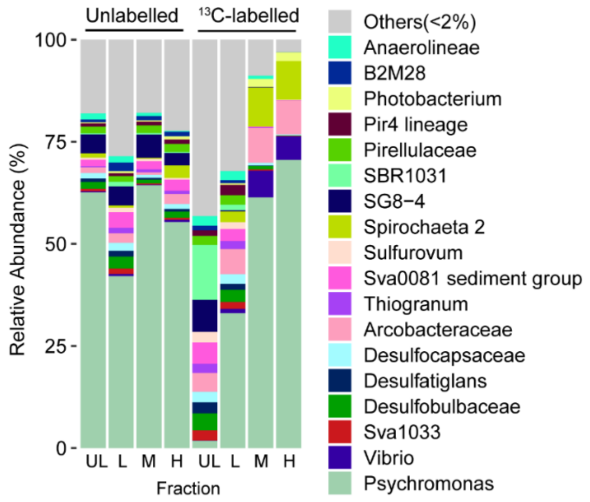

Fig. S8 Identification of glucose degraders using RNA-SIP from incubations without antibiotics (see Fig. S8 for the development of ^{13}C-CO_2 in the headspace of the SIP incubations).

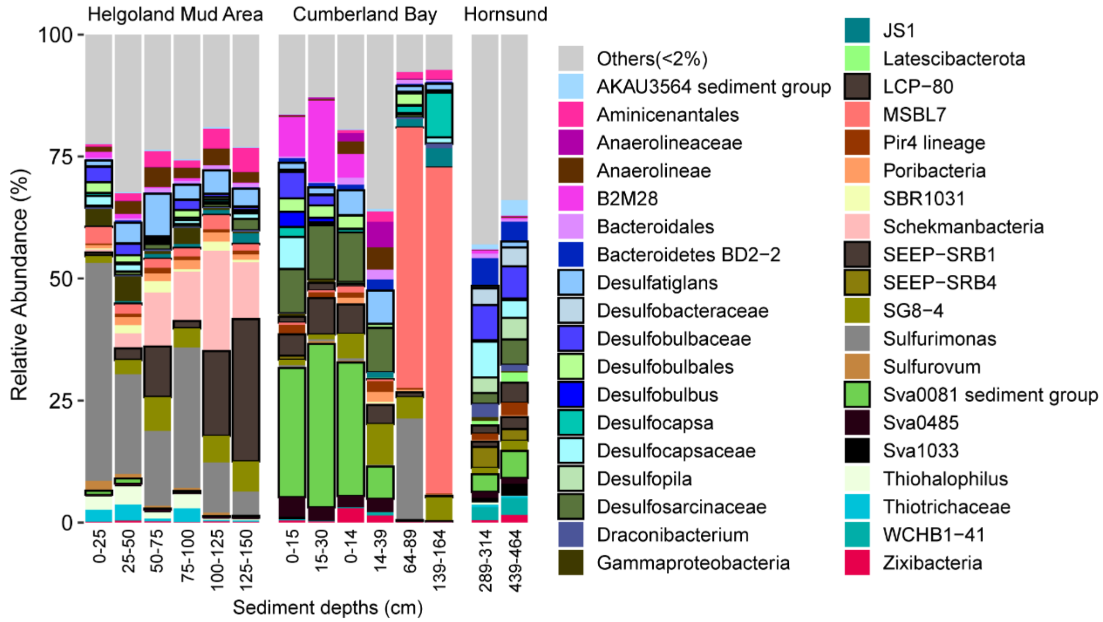

Fig. S9 Relative abundance of bacteria from original sediments based on 16S rRNA sequencing of extracted RNA. SRM are highlighted in figure.

1. Jochum LM, Schreiber L, Marshall IPG, Jorgensen BB, Schramm A, Kjeldsen KU. Single-Cell Genomics Reveals a Diverse Metabolic Potential of Uncultivated Desulfatiglans-Related Deltaproteobacteria Widely Distributed in Marine Sediment. Front Microbiol. 2018;9:2038.
